# The Relationship between Using Renin-Angiotensin System Inhibitors with Mortality of Atrial Fibrillation: A Systematic Review and Meta-Analysis

**DOI:** 10.2174/011573403X326428240902114410

**Published:** 2024-09-16

**Authors:** Reza Faramarz Zadeh, Shahab Masoumi, Negar Jafari, Venus Shahabi Rabori, Saeid Heidari-Soureshjani

**Affiliations:** 1 Cardiology, Seved-Al-Shobada Cardiology Hospital, Urmia University of Medical Sciences, Urmia, Iran;; 2 Cardiovascular Fellowship, Cardiovascular Research Center, Tabriz University of Medical Sciences, Tabriz, Iran;; 3 Cardiovascular Fellowship, Vanderbilt University Medical Center, Nashville, TN, USA;; 4 Department of Cardiology, School of Medicine, Urmia University of Medical Sciences, Urmia, Iran;; 5 International Training Fellow, Cardiology Department, Royal Albert Edvard Infirmary, Wigan, Wwl Nhs Trust, Wigan, UK;; 6 Cardiology Department, Royal Albert Edward infirmary, WWL NHS Trust, Wigan, UK;; 7 Modeling in Health Research Center, Shahrekord University of Medical Sciences, Shahrekord, Iran

**Keywords:** Renin-angiotensin system inhibitors, angiotensin-converting enzyme inhibitors, angiotensin receptor blockers, mortality, chi-square, atrial fibrillation

## Abstract

**Background:**

Atrial fibrillation (AFib) is a highly prevalent cardiac arrhythmia associated with increased mortality in affected persons. Renin-angiotensin system inhibitors (RASIs) have been suggested as potential therapeutic agents for cardiovascular and renal diseases.

**Objectives:**

However, the relationship between RASIs and mortality in AFib patients remains uncertain. Therefore, the present study was designed and implemented for this purpose.

**Methods:**

We searched PubMed/MEDLINE, Embase, Web of Science (WOS), Cochrane Library, and Scopus databases for studies published until 12 February 2024 with relevant keywords. We included studies that reported mortality outcomes in AFib patients treated with RASIs and non-users. The data extraction and quality assessment processes were conducted, and subgroup analyses and sensitivity analyses were done. The data were analyzed by Stata 15 using statistical tests, such as Chi-square and I^2^ tests.

**Results:**

A total of 15 studies (2007-2024; n=2,178,565 patients) examined the association between RASI drugs and mortality of patients with AFib. The results indicated that compared to the control group, the odds of AFib mortality in the group receiving RASIs were equal to 0.81(95% CI: 0.71-0.92; *P-*value ≤0.001). The study results did not indicate publication bias (*P-*value=0.733). During the meta-regression analysis, none of the study variables demonstrated a significant relationship with the observed heterogeneity (*P-*value > 0.20). Cumulative OR results showed that from 2022 onwards, there was enough evidence to confirm the relationship using RASIs with mortality of patients with AFib.

**Conclusion:**

Therefore, this meta-analysis suggests that the use of RASI drugs is associated with reduced AFib mortality. However, the authors emphasize the need for further high-quality studies and large-scale randomized clinical trials to validate these findings.

## 1 INTRODUCTION

Atrial fibrillation (AFib), which is the most prevalent form of cardiac arrhythmia and most frequent cardiovascular disease (CVD), is characterized by an irregular heartbeat [1]. It is also the primary cardiac cause of stroke. The likelihood of developing AFib is increased by various factors, including developing age, congenital heart disease, high blood pressure, heart or lung disease, genetic factors, neurologic disorders, endocrine disorders, smoking, and consuming excessive amounts of alcohol [2]. Due to its high morbidity and mortality, this disorder imposes a significant burden of treatment costs on the healthcare system [3]. Patients with AFib experience lower health-related quality of life (HRQoL) that negatively affects daily life and psychological health [4]. Treatments for AFib have moderate effectiveness in terms of symptom relief and functional status improvement [5]. Although catheter ablation and surgical procedures have proven to be effective in treating patients affected with AFib, they are not without their risks and potential complications. Therefore, prevention and treatment strategies in the fight against AFib should be strengthened [6].

Renin-angiotensin system inhibitors (RASIs), such as angiotensin-converting enzyme inhibitors (ACEIs) and angiotensin receptor blockers (ARBs), are widely used for hypertension treatment by affecting the renin-angiotensin-aldosterone system [7-10]. Although various studies revealed that RASIs can attenuate AFib and its recurrence [11, 12], some studies did not find signs of their preventive and promising effects in postoperative AFib, stroke, death, and hospitalization [13]. Other studies also reported that ACEIs are attributed to the incidence of AFib and risk factors for postoperative AFib [14, 15]. Therefore, due to the conflicting results of the studies and the complexity of this disease, we conducted a systematic review and meta-analysis study to investigate the relationship between using RASIs and the mortality of patients with AFib.

## 2 MATERIALS AND METHODS

### 2.1 Search Strategy

On 12/Feb/2024, an extensive literature search was performed on various electronic databases, such as PubMed/MEDLINE, Embase, Web of Science (WOS), Cochrane Library, and Scopus databases. The search strategy included keywords and Medical Subject Headings (MeSH) terms, such as ((“Angiotensin-Converting Enzyme Inhibitors” OR “Angiotensinconverting Enzyme Inhibitors” OR “ACE Inhibitors” OR “Angiotensin Converting Enzyme Inhibitor” OR “Angiotensin Receptor Antagonists” OR “Angiotensin Receptor Blockers” OR “ARBs”) AND (“atrial fibrillation” OR “auricular fibrillation” OR “AFib”) AND (“mortality” OR “death” OR “survival”)). This systematic review aimed to supplement the search with a reference list review of relevant articles and studies from the previous review. Our search was refined until we identified all publications included in the review. This assurance was achieved by developing a comprehensive search strategy, using citation tracking to review the references of the included studies to find additional relevant literature, and final searching in Google Scholar. To ensure the accuracy of our findings, we imported the peer-reviewed studies into EndNote X8 (8 November 2016, Thomson Reuters) and removed any duplicate publications.

### 2.2 Inclusion and Exclusion Criteria

We searched for clinical trials, cohort studies, case-control studies, and observational studies examining the correlation between pre and post-surgery using RASIs (ACEIs and ARBs) and all-cause mortality and survival in adult patients (≥18 years) diagnosed with AFib. Studies reported hazard ratios (HRs), odds ratios (ORs), or relative risks (RRs) with corresponding 95% confidence intervals (CIs) or provided sufficient data for their calculation. We utilized the PICO (population, intervention, control) tool to ensure consistency. The population was defined as patients with AFib, while the intervention was treatment with RASIs. We used patients who did not take RASIs and other antihypertensive medications, placebo, or no treatment as the control group. We excluded studies that did not meet these inclusion criteria, such as review articles, case series, case reports, commentaries abstract-only publications, conference publications, unpublished study protocols, letters to the editor, *in vivo* and *in vitro* studies, co-administration of RASIs with other drugs, and studies published in Non-English languages.

### 2.3 Study Selection

Two reviewers evaluated the titles and abstracts of identified studies to determine their eligibility. The full texts of studies with potential eligibility were retrieved and evaluated based on predefined criteria. Any discrepancies were resolved through discussion and consensus or by consulting a third reviewer.

### 2.4 Data Extraction

Two reviewers performed Data extraction independently based on predefined eligibility criteria and using a standardized form. The following data were extracted from each included publication: study characteristics (author, year of publication, country of the study, design, sample size), participant characteristics (sample size, age, sex, comorbidities, drugs), intervention details (type of RASIs and follow-up duration), outcomes (mortality/survival measures), quality assessment results and adjusted effect estimates OR, RR, or HR with corresponding 95% CI for patients with AFib.

### 2.5 Quality Assessment

To evaluate the quality and potential for bias in observational studies, researchers commonly use the Newcastle-Ottawa Scale (NOS) [16]. This scale is a comprehensive tool that assesses several aspects of study design, such as the selection of study participants, the comparability of groups, and the measurement of outcomes. Studies scoring at least seven on the NOS were categorized as high-quality. On the other hand, the quality and risk of bias in treatment estimates used in RCT articles were evaluated using the Jadad scale [17, 18]. The scale developed by Jadad had a scoring system that ranged from 0 to 5. Clinical trials that scored 3 points or higher were considered an indication of studies with a decent level of quality.

### 2.6 Data Synthesis and Analysis

A meta-analysis was conducted to evaluate the correlation between using RASIs and the mortality and survival of AFib. The OR was used for this analysis to indicate the effect size of the relationship between study exposure and the outcome, with a 95% CI. Random-effect models of the meta-analysis were used to calculate the overall summary estimates. Individual OR and summary estimates were graphically illustrated using forest plots. Subgroup analyses were performed based on geographical location, sample size, type of study, study period, length of follow-up, and quality assessment score as per the a priori decisions.

The researchers utilized Cochran's Q test, with a significance level of chi-square and *P* < 0.1, and the I2 statistic to assess heterogeneity across the studies. To ensure the reliability of the findings, a series of sensitivity analyses were conducted. The first analysis involved recalculating the pooled estimates after removing one study at a time to determine the impact of individual studies on summary estimates. The second analysis was a meta-regression to identify the source of differences in effect size between studies. Furthermore, Begg's and Egger's tests examined potential publication and accumulation biases. All statistical analyses were performed using Stata 14.0 (Stata LLC, College Station, TX, USA), with a significance level of *P* < 0.05.

### 2.7 Reporting Methods

The systematic review was reported based on the Preferred Reporting Items for Systematic Reviews and Meta-Analyses (PRISMA) 2020 guidelines.

### 2.8 Ethical Considerations

Since this research was conducted through a review and analysis of already published data, it did not require ethical approval. All the data utilized in this study were obtained from previously published research, and no information regarding individual patients was accessed.

## 3 RESULTS

### 3.1 Search Results

Within Fig. (**1**), the PRISMA flowchart displays our search approach. We began with an electronic search that retrieved 2953 titles/abstracts. After eliminating 242 articles with duplicate titles, we excluded an additional 18 titles/abstracts for reasons, such as non-English language [19], not containing our study indices [20-35], one study due to the use of a similar data bank, the same population, and year (with Nieuwlaat *et al*. study [36, 37].

### 3.2 Description of the Included Studies

The study comprised 15 separate investigations covering a combined population of 2,178,565 patients across several countries, such as South Africa, the United States, England, Spain, Sweden, Brazil, and China [7, 36, 38-50]. The studies were conducted between 2007 and 2024, and the patients were followed up for a period ranging from 1 to 96 months (Table **1**). Tables **2** and **3** also show descriptive characteristics related to underlying diseases, such as the use of substances, medications, and adjusted variables in the studied patients.

### 3.3 Relationship between using RASIs with Mortality of Patients with AFib

**Fig. (2):**
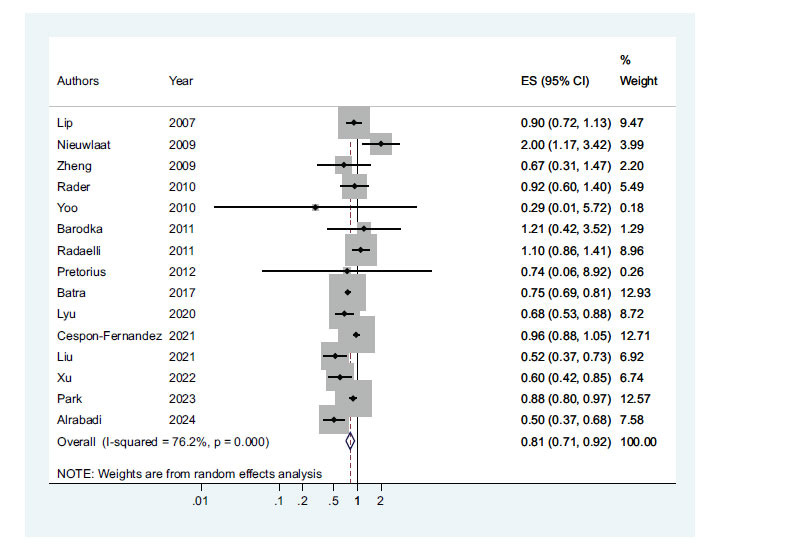
Relationship using RASIs with mortality of patients with AFib.

In the examination of the relationship between using RASIs and the mortality of patients with AFib, it was observed that compared to the control group, the odds of AFib mortality in the group receiving RASIs were equal to 0.81(95% CI: 0.71-0.92; *P-*value ≤0.001). Therefore, it can be said that receiving AFib leads to a decrease in the chance of mortality (Fig. **2**).

### 3.4 Assessment of Publication Bias

In the study examining the relationship between the use of RASIs and mortality in AFib, no publication bias was observed based on the results of Egger's test (*P-*value=0.733) and Baggs' test (*P-*value=0.921). The corresponding funnel diagram can be found in Fig. (**3**).

### 3.5 Meta-Regression and Sensitivity Analysis

**Fig. (4):**
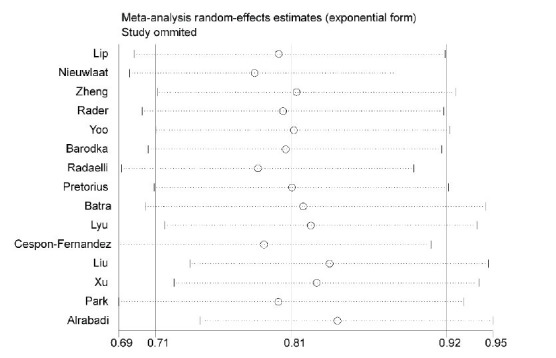
Sensitivity analysis in relation to using RASIs with mortality of patients with AFib.

During the meta-regression analysis, none of the study variables demonstrated a significant relationship with the observed heterogeneity (*P-*value > 0.20). Furthermore, the sensitivity analysis did not reveal significant changes in the study results (Fig. **4**).

### 3.6 Subgroup Analysis

**Fig. (5):**
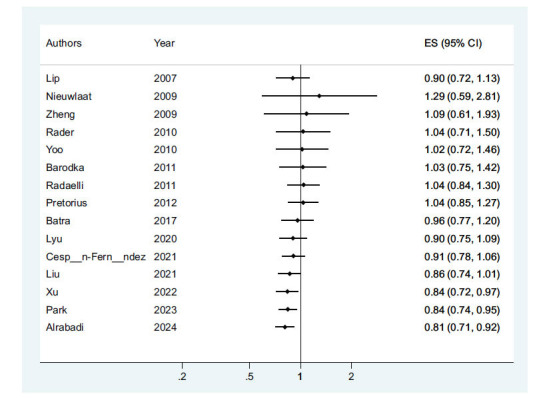
The cumulative OR in different years.

According to the subgroup analysis, the odds ratio of mortality for AFib varied in different studies. In American and Canadian studies, the odds ratio was 0.83 (0.54-1.29), while in European studies, it was 0.95 (0.76-1.17). For studies conducted in Asia, the odds ratio was 0.68 (0.54-0.86). The odds ratio also varied based on the study design, with retrospective studies at 0.88 (0.71-1.10), cohort studies at 0.74 (0.61-0.90), and RCTs at 0.74 (0.06-8.92). Studies conducted in 2011 or earlier had an odds ratio of 1.04 (0.83-1.30), while those conducted in 2012 or later had an odds ratio of 0.72 (0.62-0.84). Cumulative OR results showed that from 2022 onwards, there is enough evidence to confirm the relationship using RASIs with mortality of patients with AFib (Fig. **5**).

The sample size and average age of the participants also affected the odds ratio, with smaller sample sizes and older ages having lower odds ratios. Follow-up time also played a role, with studies with less than 12 months of follow-up having an odds ratio of 0.61 (0.51-0.72) and those with more than 12 months having an odds ratio of 0.82 (0.71-0.94). Finally, the quality assessment score of the study also had an impact, with good-quality studies having an odds ratio of 0.64 (0.54-0.76), moderate quality studies at 0.92 (0.86-0.98), and low-quality studies at 1.36 (0.83-2.22) (Table **4**).

## 4 DISCUSSION

This meta-analysis aimed to investigate the association between using RASIs with mortality and survival of AFib. To the best of our knowledge, a systematic review and meta-analysis study has not yet been done in this regard except for one meta-analysis that had a limited study of subgroup analysis of death. Therefore, we discuss and review similar studies here. Our study revealed that compared to the control group, the odds of AFib mortality in the group receiving RASIs were equal to 0.81(95% CI: 0.71-0.92; *P-*value ≤0.001). In line with our study, Chaugai *et al*. revealed in their meta-analysis that RASIs have protective effects against heart failure and cardiovascular events in AFib patients. They reduced the incidence of heart failure by 14% (OR= 0.86, 95% CI: 0.76–0.97, *P*=0.018) and decreased the risk of cardiovascular events by 17% (OR= 0.83, 95% CI: 0.70–0.99) in AFib patients [12]. Another study reported that the use of ACEIs and ARBs has promising effects for preventing AF in individuals with heart failure. However, their effectiveness differs for hypertensive patients with normal left ventricular function. Additionally, the beneficial use of ACEIs or ARBs for preventing AF after electrical cardioversion has been discovered only for secondary prevention [51]. In their meta-analysis, Jia *et al*. and Ma indicated that RASIs can effectively prevent AFib recurrence and persistent AFib [11, 52]. Another study demonstrated that early administration of ACEIs reduced the risk of cardiac rupture by 33% (RR= 0.67, 95% CI: 0.50–0.90, *p*= 0.008) after acute myocardial infarction [53].

The consistent findings of previous studies suggest a potential beneficial effect of RASIs on mortality in AFib patients. It also highlights the biological plausibility of the observed association between RASIs and reduced mortality due to AFib, citing the known physiological mechanisms of these drugs in blocking the angiotensin II pathway, which may lead to favorable cardiovascular outcomes in patients with AFib [54]. The accumulation of epicardial adipose tissue may be influenced by RASIs, which could prevent inflammation and serve as a viable therapeutic option for preventing AF. This approach may be particularly beneficial for patients suffering from heart failure and those with known left ventricular dysfunction [55]. The RASIs may have a critical role in causing AFib by affecting various processes, such as inflammatory responses, cardiac electrical remodeling, inhibiting oxidative stress, and the buildup of epicardial fat [56]. In particular, the ACE/AII/AT1 axis can increase inflammation by promoting oxidative stress, cytokine release, reactive oxygen species (ROS) production, and macrophage phenotype switching. This axis can contribute to electrical cardiac remodeling through these mechanisms, resulting in morphological changes in atrial cardiomyocytes. Moreover, it may encourage the accumulation and inflammation of epicardial fat that may influence the onset of AFib, either directly or indirectly [55, 57, 58].

In contrast with our results, a meta-analysis found no additional benefit of RASIs in reducing the risk of postoperative AFib (OR= 1.04; 95% CI, 0.91-1.19; *P =* 0.55) and death (OR= 1.07, 95% CI: 0.85-1.35, *P =* 0.56) in the patients undergoing cardiac surgery [13]. A review study by Nair *et al*. showed that using RASIs alone did not prove effective in reducing AFib recurrences after cardioversion unless used with antiarrhythmic therapy. Additionally, using RASIs alone failed to prevent AFib recurrences in individuals who underwent catheter ablation [59].

Conflicting results exist regarding the use of RASIs and their impact on mortality and complications in patients undergoing cardiac surgery [60]. Our study adds to the evidence on RASIs and mortality in AFib. However, differences in study populations, designs, demographics, comorbidities, medication dosages, and follow-up durations could affect findings. Despite efforts to control confounding variables, residual confounding and selection bias could still exist, impacting the observed relationship between RASIs and mortality in AFib patients. So, more clinical and observational studies are needed to draw more reliable results.

## 5 LIMITATIONS

It is important to note that our study has some limitations. The lack of reporting the dosage of RASI in the included studies was a limitation of our study. Secondly, the quality and completeness of the available data varied across studies. In some studies, most of the confounding variables were not controlled, which could have affected the accuracy and reliability of our findings. Although we conducted a comprehensive systematic review, we had to exclude some relevant studies based on our exclusion criteria, which might have impacted the comprehensiveness of our analysis. Finally, as with any observational study, we cannot infer causality, and further prospective studies are necessary to validate our findings and determine the underlying mechanisms.

## CONCLUSION

Our systematic review and meta-analysis show a promising link between using RASIs and reduced mortality among individuals with AFib. Cumulative OR results showed that from 2022 onwards, there was enough evidence to confirm this relationship. These findings highlight the potential therapeutic benefits of these drugs in improving outcomes for patients with this common cardiac condition. However, more studies controlling the most critical factors involved in the occurrence of AFib are needed to obtain more robust results.

## Figures and Tables

**Fig. (1):**
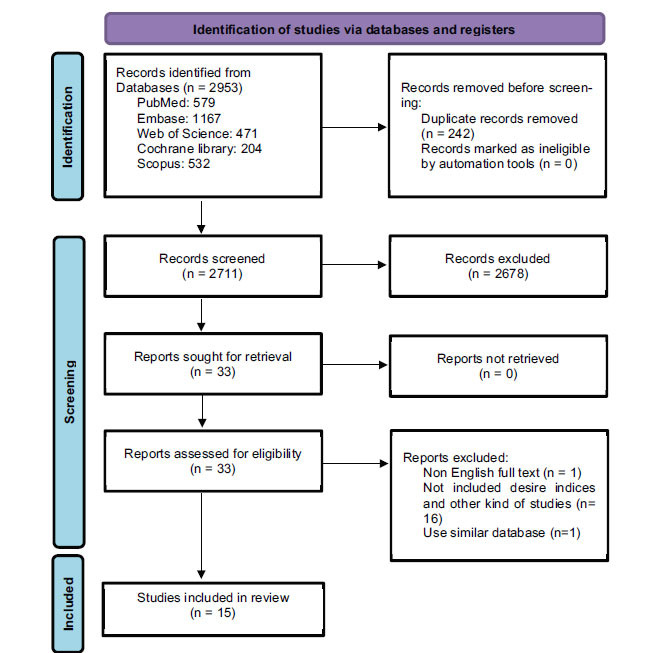
PRISMA Flow diagram of the steps for including studies in the meta-analysis.

**Fig. (3):**
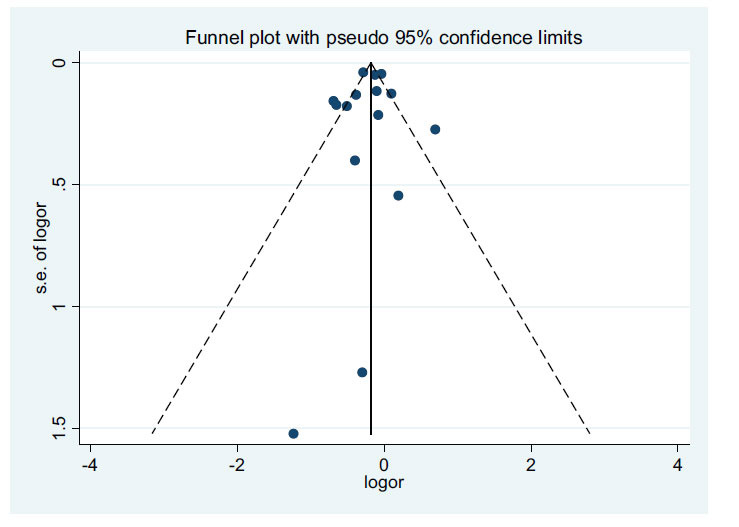
Assessment of publication bias in relation to using RASIs with mortality of patients with AFib.

**Table 1:** The key features of the studies included in the meta-analysis.

**Authors**	**Year**	**Country**	**Study Type**	**Type of Drugs**	**N all Cases**	**N of Causes: RASIs/Control**	**Gender (Male) RASIs/Control (%)**	**Age RASIs/ Control (Mean)**	**Follow-up (Months)**	**OR**	**QA**
Lip [38]	2007	Sweden	Retrospective	ACEIs/ARBs	7,329	4760/2569	71/66	70.2/72.3	18.7	0.9(0.72-1.13)	8
Nieuwlaat [36]	2009	UK	Retrospective	ACEIs	6,104	3,052/3,052	80.5/80.7	64.9/64.8	In hospital	2(1.17-3.42)	7
Zheng [39]	2009	China	Retrospective	ACEIs/ARBs	139	45/94	37/64	55.4/55.6	14.6	0.67(0.31-1.47)	8
Rader [40]	2010	USA	Retrospective	RASIs	6,874	3437/3437	72.4/74.8	66/66	In hospital	0.92(0.60-1.40)	8
Yoo [7]	2010	Sought Korea	Retrospective	RASIs	472	296/ 176	63.9/56.8	65/65	1	0.29(0.005-5.72)	9
Barodka [41]	2011	USA	Retrospective	RASIs	346	122/224	71/62	73.8/74.5	1	1.21(0.42-3.52)	9
Radaelli [42]	2011	Brazil	Retrospective	RASIs	3139	1635/1504	64.2/68.9	61.2/61.6	In hospital	1.10(0.87-1.41)	7
Pretorius [43]	2012	USA	RCT	Ramipril	445	298/147	67.7/64.0	58.9/60.0	In hospital	0.74(0.08-8.92)	5
Batra [44]	2017	Sweden	Retrospective	ACEIs/ARBs	112648	83291/29357	66.1/60	71/74	36	0.75(0.70-0.81)	9
Lyu [45]	2020	China	Cohort	ACEIs/ARBs	1,991	759/1,232	46/44.6	72/71	12	0.68(0.53-0.88)	9
Cespón-Fernández [46]	2021	Spain	Cohort	ACEIs/ARBs	9365	4885/4480	60.3/60.2	83.0/83.2	44.4	0.96(0.87-1.05)	8
Liu [47]	2021	China	Retrospective	ACEIs/ARBs	734	410/324	32.68/34.24	50.0/51.7	70.8	0.52(0.37-0.73)	9
Xu [48]	2022	China	Cohort	ACEIs/ARBs	1110	574/536	45.8/42.7	73/74	12	0.60(0.43-0.85)	9
Park [49]	2023	Sought Korea	Cohort	ACEIs/ARBs	2,025,849	1329179/ 696,670	57.75/43.9	56.45/57.4	96	0.88(0.80-0.97)	8
Alrabadi [50]	2024	Jordan	Prospective multicenter observational	ACEIs/ARBs	2020	788/1232	44.9/46.7	69.8/69.7	12	0.50(0.37-0.68)	9

**Abbrevations:** N; Number; RASIs; Renin-angiotensin system inhibitors; ACEIs; Angiotensin-converting enzyme inhibitors, ARBs; Angiotensin receptor blockers, OR; Odd ratio, QA; quality assessment, RCT; Randomized clinical trial.

**Table 2:** Records of underlying diseases using substances and medications.

**Authors**	**Year**	**Primary HTN RASIs/Control (%)**	**Diabetes RASIs/Control (%)**	**Prior MI RASIs/Control (%)**	**BMI (Mean) RASIs/Control (%)**	**Smoking RASIs/Control (%)**	**Statins usage RASIs/Control (%)**	**Diuretics RASIs/Control (%)**	**β-blocker RASIs/Control (%)**	**Calcium channel blocker RASIs/Control (%)**	**Aspirin RASIs/Control (%)**	**Anticoagulant RASIs/Control (%)**	**Digoxin RASIs/Control (%)**
Lip [38]	2007	85/62	27/17	19/24	29.6/27.9	NR	NR	NR	NR	NR	NR	NR	NR
Nieuwlaat [36]	2009	67.8/68.7	14.4/14.1	46.4/46	NR	51.1/50.2	NR	NR	NR	NR	NR	NR	NR
Zheng [39]	2009	71.11/21.27	6.66/4.25	NR	NR	NR	NR	NR	NR	NR	NR	NR	NR
Rader [40]	2010	86/70	45/30	NR	29/28	66/65	NR	NR	68/66	24/29	NR	NR	NR
Yoo [7]	2010	68.6/61.4	43.6/31.8	NR	25/24	NR	NR	18.2/9.1	69.3/64.8	40.5/58.5	84.8/81.8	NR	NR
Barodka [41]	2011	90.2/75.9	32.8/27.2	NR	27.5/27.2	21.9/27.3	NR	NR	81.2/74.6	NR	80.3/75.5	NR	7.6/6.8
Radaelli [42]	2011	83.2/60.9	34.3/24.5	46.9/36.2	NR	31.0/38.2	NR	NR	NR	NR	NR	NR	NR
Pretorius [43]	2012	61.1/66.0	22.1/20.4	NR	29.2/30.1	10.6/16.3	53.6/57.1	14.55/18.7	50.3/49.0	19.2/18.4	NR	NR	NR
Batra [44]	2017	64.7/46.2	26.6/16.9	24.1/26.7	27/25	24.1/23.8	88.3/73.1	34.1/30.2	91.0/83.8	16.1/14.8	93.6/90.5	7.5/5.7	3.1/3.5
Lyu [45]	2020	75.6/43.5	19.8/12.9	20.3/17.9	23.7/23.2	24.6/19.3	39.5/14.3	55.5/28.2	54.0/37.7	28.5/19.9	70.0/45.5	24.4/21.0	40.4/24.5
Cespón-Fernández [46]	2021	65.1/62.4	20.1/18.6	8.6/7.7	29.6/29.5	NR	39.3/37.6	NR	37.0/35.8	3.2/4.2	NR	81.7/81.2	15.6/14.9
Liu [47]	2021	26.8/20.2	22/23	86.4/88.3	NR	10.9/12.5	9.3/8.9	65.4/63.8	34.6/34.2	8.2/6.2	NR	61.9/56.8	56.0/60.7
Xu [48]	2022	NR	19.2/24.8	40.8/26.1	24.0/23.6	19.6/23.2	44.1/20.5	49.1/26.1	54.2/47.8	35.2/39.2	70.6/51.3	13.8/12.7	49.1/26.1
Park [49]	2023	NR	ACEIs: 34.0 ARBs: 24.4 Control: 14.1	NR	NR	ACEIs: 20.0 ARBs: 20.3 Control: 15.9	ACEIs: 30.4 ARBs: 26.0 Control: 20.4	NR	NR	NR	ACEIs: 29.6 ARBs: 27.2 Control: 37.4	NR	NR
Alrabadi [50]	2024	89.2/65.1	48.4/40.5	27.2/21.7	NR	10.7/16	NR	NR	NR	NR	NR	NR	NR

**Abbreviations:** HTN; Hypertension, MI, Myocardial infraction; BMI; Body mass index, RASIs; Renin-angiotensin system inhibitors; ACEIs; Angiotensin-converting enzyme inhibitors, ARBs; Angiotensin receptor blockers, NR; Not reported.

**Table 3:** Adjusted variables in studies included in the meta-analysis.

**Authors**	**Year**	**Adjusted Variables**
Lip [38]	2007	1, 6, 7
Nieuwlaat [36]	2009	NR
Zheng [39]	2009	NR
Rader [40]	2010	1, 2, 3, 6, 7, 13
Yoo [7]	2010	1, 2, 3, 6, 7, 9, 11.
Barodka [41]	2011	1, 2, 3, 4, 6, 7, 8, 9
Radaelli [42]	2011	1
Pretorius [43]	2012	NR
Batra [44]	2017	1, 2, 6, 7, 8, 9, 10, 12
Lyu [45]	2020	1, 2, 3, 4, 6, 7, 8, 9
Cespón-Fernández [46]	2021	NR
Liu [47]	2021	1, 2, 4, 5, 6, 7, 8
Xu [48]	2022	1, 3, 6, 7, 8
Park [49]	2023	1, 2, 3, 4, 5, 7, 8
Alrabadi [50]	2024	1, 2, 6, 7

**Note:** 1; Age, 2; Sex, 3; Body mass index, 4; Smoking, 5; Alcohol consumption, 6; Cardiovascular diseases, 7; Comorbidities, 8; Other drugs intake, 9; Creatinine concentration, 10; Duration of hospitalization, 11; Red blood cell transfusion, 12; Killip class on admission, 13; Race.

**Table 4:** Subgroup analysis in relation to using RASI with mortality of patients with AF.

**Characteristics**		**Number of Studies**	**RR (95% CI)**	***P-*value**
Study location	America and Canada	5	0.83(0.54-1.29)	0.418
	Europe	4	0.95(0.76-1.17)	0.609
	Asia	6	0.68(0.54-0.86)	0.001
Study design	Retrospective	10	0.88(0.71-1.10)	0.270
	Cohort	4	0.74(0.61-0.90)	0.002
	RCTs	1	0.74(0.06-8.92)	0.813
Period of time	2011 or earlier	7	1.04(0.83-1.30)	0.721
	2012 or later	8	0.72(0.62-0.84)	0.001
Sample size	<1000	5	0.57(0.43-0.77)	0.001
	≥1000	10	0.84(0.73-0.96)	0.009
Age average	<70	9	0.83(0.63-1.08)	0.157
	≥70	6	0.80(0.68-0.94)	0.006
Follow up time	≤ 1 years	5	0.61(0.51-0.72)	0.001
	˃1 years	6	0.82(0.71-0.94)	0.004
	Non-reported	4	1.19(0.86-1.65)	0.303
Quality assessment	Good quality	7	0.64(0.54-0.76)	0.001
	Moderate quality	5	0.92(0.86-0.98)	0.008
	Low quality	3	1.36(0.83-2.22)	0.220

## Data Availability

All data generated or analysed during this study are included in this published article.

## LIST OF ABBREVIATIONS

ACEIs: Angiotensin-converting Enzyme Inhibitors
AFib: Atrial Fibrillation
ARBs: Angiotensin Receptor Blockers
BMI: Body Mass Index
CI: Confidence Interval
CVD: Cardiovascular Disease
HRQoL: Health-related Quality of Life
HRs: Hazard Ratios
HTN: Hypertension
MI: Myocardial Infraction
N: Number
NOS: Newcastle-ottawa Scale
NR: Not Reported
OR: Odds Ratio
PRISMA: Preferred Reporting Items for Systematic Reviews and Meta-Analyses
QA: Quality Assessment
RASIs: Renin-angiotensin System Inhibitors
RCT: Randomized Clinical Trial
ROS: Reactive Oxygen Species
RR: Relative Risks

## SUPPLEMENTARY MATERIAL

PRISMA checklist as Supplementary material is available on the publishers' website along with the published article.
